# Conductive Response Imaging in Thigh Muscle Compartments by Electrical Impedance Tomography for Efficient Bicycle Training Strategy

**DOI:** 10.1007/s10439-026-03988-z

**Published:** 2026-01-22

**Authors:** Daichi Furukawa, Kiagus Aufa Ibrahim, Tomoyuki Shirai, Masahiro Takei

**Affiliations:** 1 Department of Mechanical Engineering, Graduate School of Science and Engineering, Chiba University, Chiba, 263-8522 Japan; 2 MTG Company Ltd., Nagoya-shi, Aichi 453-0041 Japan

**Keywords:** Electrical impedance tomography, Bicycle training, Electrical muscle stimulation, Thigh muscle, Blood lactate concentration

## Abstract

The conductive response in thigh muscle compartments has been imaged by electrical impedance tomography (EIT) under bicycle-operation conditions and muscle-stimulation conditions to determine an efficient training strategy. EIT is already applied to evaluate the effectiveness of electrical muscle stimulation (EMS) on human muscle, and hybrid of EMS which combines EMS on biceps and voluntary resistance training simultaneously. In this study, we newly applied EIT to the thigh muscle compartments and imaged the conductive response under bicycle-operation conditions and muscle-stimulation conditions. The bicycle-operation conditions are high pedal rate in low torque (HPLT) and low pedal rate in high torque (LPHT). The muscle-stimulation conditions are bicycle condition and EMS-combined bicycle condition. As a result, EIT successfully imaged the conductive response in three muscle compartments which are T1 compartment (quadriceps), T2 compartment (hamstrings), and T3 compartment (other than the quadriceps and hamstrings). The spatial-mean conductivity changes $\Delta \langle {{\boldsymbol{\sigma}}}_{{\boldsymbol{P}}}\rangle$ (the power of bicycle training ${\boldsymbol{P}}=103,152,\mathbf{o}\mathbf{r}194[\mathbf{W}]$) increased with increasing power ${\boldsymbol{P}}$ of the bicycle training across all four conditions and in all muscle compartments (${\boldsymbol{\beta}}=0.531,\boldsymbol{ }{\boldsymbol{p}}<0.001$). The $\Delta \langle {{\boldsymbol{\sigma}}}_{{\boldsymbol{P}}}\rangle$ of HPLT tended to be larger than $\Delta \langle {{\boldsymbol{\sigma}}}_{{\boldsymbol{P}}}\rangle$ of LPHT (${\boldsymbol{\beta}}=-35.52,\boldsymbol{ }{\boldsymbol{p}}<0.001$). The $\Delta \langle {{\boldsymbol{\sigma}}}_{{\boldsymbol{P}}}\rangle$ of EMS-combined bicycle condition tended to be larger than $\Delta \langle {{\boldsymbol{\sigma}}}_{{\boldsymbol{P}}}\rangle$ of bicycle condition (${\boldsymbol{\beta}}=12.85,{\boldsymbol{p}}<0.05$). The key findings are that HPLT and EMS-combined bicycle condition are a more efficient bicycle training than LPHT and bicycle condition for increasing $\Delta \langle {{\boldsymbol{\sigma}}}_{{\boldsymbol{P}}}\rangle$.

## Introduction

BICYCLE training for both young and elderly individuals helps reduce the muscle loss that occurs with skeletal muscle disorders [1], such as muscular dystrophy [2], neuromuscular conditions [3], and sarcopenia [4]. Bicycle training is widely utilized in sports clubs, geriatric health facilities, and ordinary households because bicycle training is less likely to cause knee or hip joint disorders [5]. Thigh muscle compartments, such as quadriceps, hamstrings, and adductors, play a crucial role in the pedaling motion [6]. Efficient bicycle training is determined by a visual evaluation of the response of the thigh muscle compartments. Previous research has examined the physiological responses associated with distinct bicycle-operation conditions, particularly contrasting a high pedal rate with low torque (HPLT) and a low pedal rate with high torque (LPHT). HPLT results in higher heart rate (HR), mean arterial pressure (MAP), ${\mathrm{O}}_{2}$ uptake (${\dot{\mathrm{V}}\mathrm{O}}_{2}$), ${\mathrm{CO}}_{2}$ emission (${\dot{\mathrm{V}}\mathrm{CO}}_{2}$), and tissue hemoglobin index (THI) under ventilatory threshold intensity [5]. In addition, comparisons of muscle-stimulation conditions, bicycle condition and electrical muscle stimulation (EMS)-combined bicycle condition, have also become necessary. Recently, EMS, which stimulates the thigh and hip muscles [7], has been proposed as an efficient training strategy to help strengthen these specific muscle groups without causing joint strain [8]. Unfortunately, EMS training alone produces variable or inconsistent results [9]. In this regard, EMS-combined bicycle, which combines bicycle condition with simultaneous EMS of the quadriceps, hamstrings, and gluteus maximus muscles, has been proposed to enhance muscle strength in the thighs and hips effectively with a lower effort [10]. EMS has been shown to activate cycling muscles effectively, stimulating lower limb muscle groups including both agonist and antagonist muscles, and potentially supporting cycling through induced muscle contractions [11]. However, the lack of a direct observation tool for the response of the thigh muscle compartments under bicycle-operation conditions and muscle-stimulation conditions highlights the need for real-time imaging techniques.

Generally, magnetic resonance imaging (MRI), computed tomography (CT), ultrasound imaging (UI), and near-infrared spectroscopy are utilized to image the physiological response of the thigh muscle compartments [12]. MRI provides excellent soft tissue contrast and high-resolution imaging of skeletal muscle mass [13]. CT provides clear images of muscle geometry to accurately distinguish muscle, fat, and bone [14]. Portable UI conducts rapid measurement for the evaluation of muscle thickness muscle fiber plume angle, and muscle bundle activity [15, 16]. Wearable NIRS has the ability to measure skeletal muscle oxygenation while exercising [17]. At the same time, MRI and CT cannot be used during bicycle training due to their large size and lack of portability [18]. In addition, MRI and CT are expensive and complicated to operate. Also, CT has the risk of ionizing high radiation exposure [13] while UI is unable to simultaneously image the physiologically induced conductive response in multiple muscle compartments due to the limited field of view to image the muscle structure [18]. NIRS also has limitations in deeper tissue imaging [19, 20]. Under these circumstances, these conventional imaging methods have drawbacks in achieving real-time imaging of the response of thigh muscle compartments under bicycle-operation conditions and muscle-stimulation conditions.

Since electrical signals directly represent the physiological response of muscle activity, surface electromyography (sEMG) and electrical impedance measurement are utilized as an alternative to address the limitations of the conventional muscle response imaging techniques [21]. The electrical signal presents a promising approach to achieve real-time measurement to evaluate the conductive response of the thigh muscle under bicycle-operation conditions and muscle-stimulation conditions. The time-frequency signal of the sEMG quantitatively reflects the functional state of the muscle [22], muscle group coordination, and muscle strength [23]. Electrical impedance measurement assesses the impedance changes of the finger flexor muscle during isometric contraction [24] to evaluate the forearm muscle with high reliability [25] and to distinguish the intracellular structure of slow-twitch and fast-twitch muscle fibers [26]. However, sEMG is associated with increased complexity and signal crosstalk, making it insufficiently reliable for evaluating the effects during bicycle training [27]. Also, conventional electrical impedance measurements are inadequate for imaging conductive responses in specific thigh muscle compartments under bicycle-operation and muscle-stimulation conditions since conventional electrical impedance measurements lack sufficient location specificity to localize the region/depth being measured accurately [28].

In order to overcome the inadequacies of sEMG and conventional electrical impedance measurement methods, electrical impedance tomography (EIT) is of interest. EIT is a non-invasive, non-ionizing, harmless, low-cost, easy to operate, and capable of real-time tomographic imaging [29]. Recent progress of real-time EIT utilized a robust algorithm called target adaptive differential iterative reconstruction for real-time bedside monitoring in clinical settings [30]. Recent machine learning EIT reconstruction methods have employed 1D-CNN to address the inverse problem, thereby facilitating enhanced reconstruction outcomes for complex geometries [31]. We already applied EIT to evaluate the effectiveness of EMS on human calf muscles [14]. We also applied EIT to evaluate the physiological-induced conductive response in arm muscle compartments under *hybrid*EMS [32], which combines EMS stimulation of the biceps with voluntary resistance training simultaneously [10]. Therefore, in this study, we newly applied EIT to the thigh muscle compartments and imaged the conductive response under bicycle-operation conditions and muscle-stimulation conditions.

The objectives of this study are (1) to apply EIT to image the conductive response in thigh muscle compartments under bicycle-operation conditions and muscle-stimulation conditions, (2) to investigate the effect of bicycle training under bicycle-operation conditions and muscle-stimulation conditions based on EIT, and (3) to quantitatively evaluate the spatial-mean conductivity of the conductive response in thigh muscle compartments under bicycle-operation conditions and muscle-stimulation conditions.

## Materials and Methods

### Experimental Setup

**Fig. 1:**
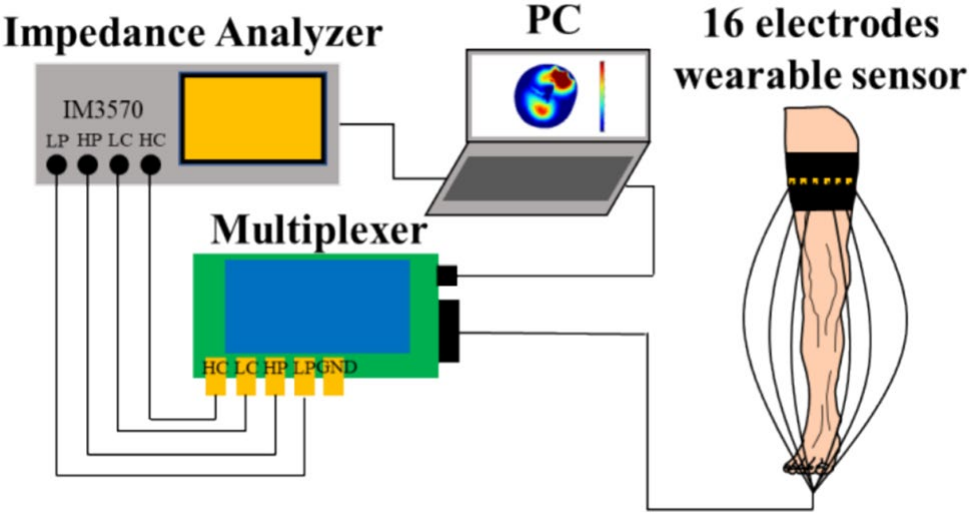
EIT system

**Fig. 2:**
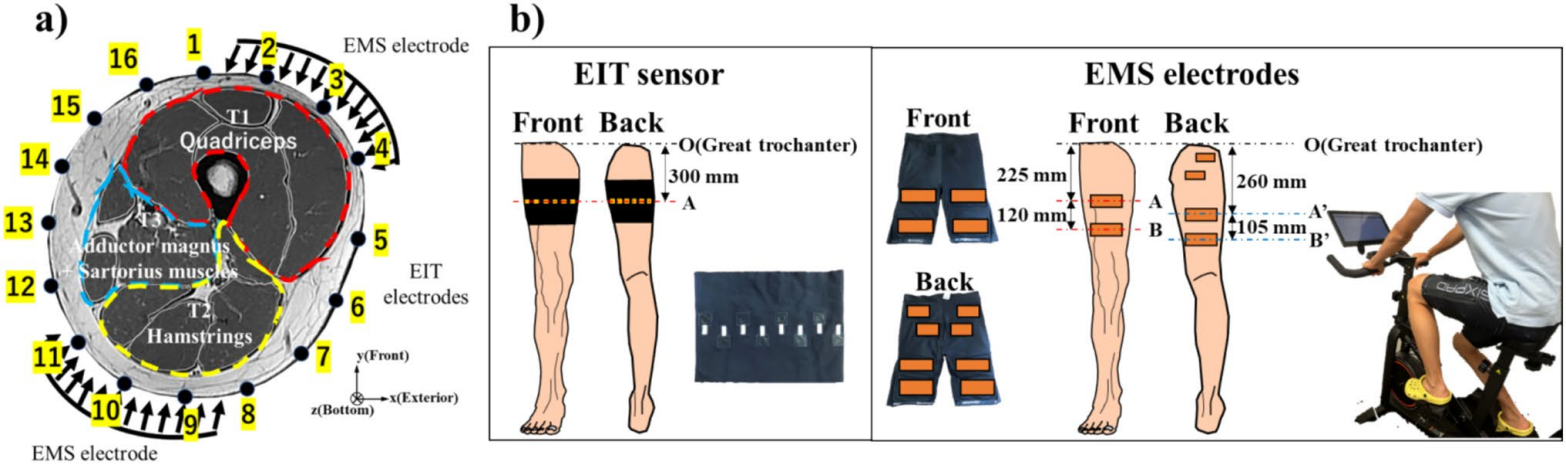
**a** The muscle compartments of the right thigh. **b** The location of EIT sensor and EMS electrodes

Fig. 1 shows the EIT system [14] which consists of an impedance analyzer (IM3570, HIOKI, Japan), a digital multiplexer (made by Takei lab based on Arduino Due), a wearable sensor [33] to which sixteen dry electrodes are attached, and a PC on which image reconstruction algorithm software is installed. The impedance analyzer injects 1 mA sinusoidal current into the electrodes by an adjacent current injection method [34]. The impedance analyzer measures the impedance via a digital multiplexer. The impedance analyzer has an impedance measurement accuracy of 0.08% [34]. The impedance analyzer covers excitation frequencies from 4 Hz to 5 MHz [35]. Fig. 2a shows the position of EIT electrodes and EMS electrodes. The number from 1 to 16 indicates the location of each EIT electrode. Fig. 2b shows the location of the EIT sensor and EMS electrodes.

### Experimental Method

The Jacobian matrix is defined as $\mathbf{J}=[{\mathbf{J}}_{1}, \dots , {\mathbf{J}}_{n},\dots , {\mathbf{J}}_{N}]\in {\mathbb{R}}^{M\times N}$, where ${\mathbf{J}}_{n}={\left[{J}_{1}, \dots , {J}_{m},\dots , {J}_{M}\right]}^{\mathrm{T}}\in {\mathbb{R}}^{M}$, $m$ is the measurement number ($1\le m\le M$), $n$ is the mesh element number ($1\le n\le N$) [14]. The Jacobian matrix element ${J}_{mn}$ of the $m$-th measured voltage pattern at the $n$-th mesh element [33] is expressed by [14]

1 $$\begin{array}{c}{J}_{mn}=\frac{\partial {v}_{m}}{\partial {\sigma }_{n}}={\int }_{\Omega }\nabla u\left({i}^{e}\right)\bullet \nabla u\left({i}^{m}\right)d\Omega \#\end{array}$$

where $\nabla u\left({i}^{e}\right)$ is the gradient of potential produced by injecting current $i$ into the *e*-th electrode, $\nabla u\left({i}^{m}\right)$ is the potential produced by injecting current $i$ into the $m$-th measured voltage pattern, ${v}_{m}$ is the measured voltage at the $m$-th measured voltage pattern, ${\sigma }_{n}$ is the conductivity at the $n$-th mesh, and $\Omega$ is the electrical field area inside the EIT sensor [14].

The image reconstruction algorithm to reconstruct ${\boldsymbol{\sigma}}$ from resistance $\mathbf{R}$ uses the Gaussian–Newton method [36] expressed by

2 $$\begin{array}{c}{{\boldsymbol{\sigma}}}^{k+1}={{\boldsymbol{\sigma}}}^{k}+{\left({\mathbf{J}}^{\mathrm{T}}\mathbf{J}+\uplambda \mathbf{I}\right)}^{-1}{\mathbf{J}}^{\mathrm{T}}\Delta R\#\end{array}$$

where $\mathbf{I}$ is a regularization matrix based on Tikhonov regularization, which is an identity matrix [37], $\uplambda$ is a regularization factor which was determined automatically by the L-curve method [38]. $\Delta \mathbf{R}=\left[\Delta {R}_{1}, \dots ,\Delta {R}_{m}, \dots ,\Delta {R}_{M}\right]\in {\mathbb{R}}^{M}$ is the resistance difference which is expressed by

3 $$\begin{array}{c}\Delta {R}_{m}\left(t\right)={R}_{m}\left(t\right)-{R}_{m}\left(0\right)\#\end{array}$$

where ${R}_{m}\left(0\right)$ is the initial measured resistance and ${R}_{m}\left(t\right)$ is the resistance at time $t$.

### Experimental Condition

**Fig. 3:**
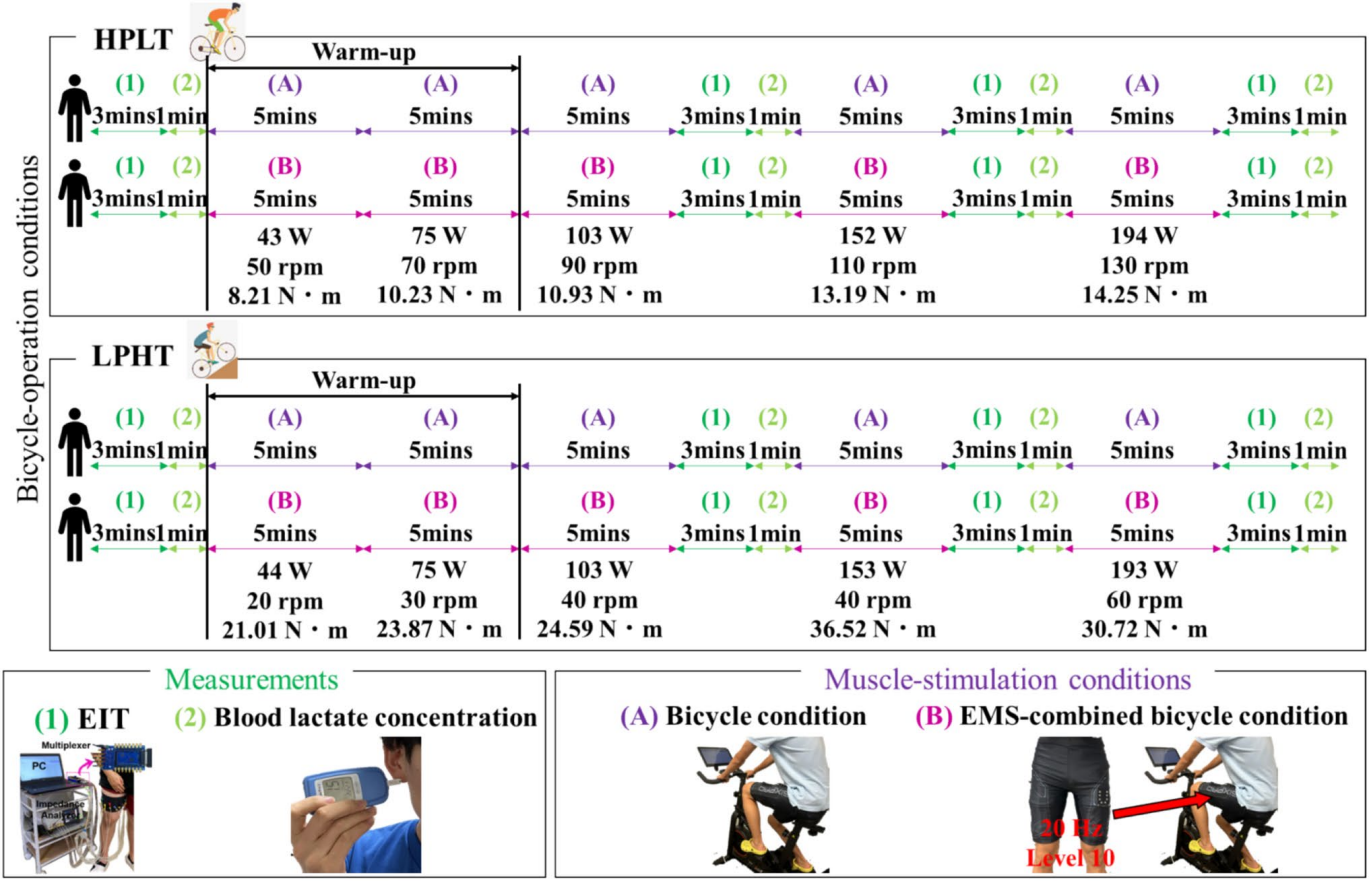
Experimental protocol

Seven healthy men (age: 27.5 ± 6.5 years, height: 175.0 ± 8.0 cm, skeletal muscle mass: 28.6 ± 6.5 kg) volunteered for this study. Fig. 3 shows the experimental protocol. We defined efficient bicycle training as bicycle training that elicits a greater conductive response in the stimulated muscles with the same power of the bicycle training. The general exercise conditions are 10 min of warm-up bicycle training and 15 min of bicycle training while power $P$ increases every 5 min. The power $P$, pedal rate, and torque of each bicycle training are shown in Fig. 3. In order to determine an efficient training strategy, bicycle training is performed under bicycle-operation conditions and muscle-stimulation conditions. The bicycle-operation conditions are HPLT and LPHT. The muscle-stimulation conditions are bicycle condition and EMS-combined bicycle condition. EMS-combined bicycle condition combines bicycle condition with EMS of the quadriceps, hamstrings, and gluteus maximus muscle, which is always performed during the bicycle condition. Electrical stimulation creates stimulation to the voluntary muscle contractions during cycling, potentially enhancing the training effect [39]. The EMS device (SIXPAD Powersuit Bottom, MTG Co., Ltd, Japan) has a constant current control, although the voltage values vary. Electrical stimulation was applied intermittently, alternating between stimulation and pause phases, with a stimulation frequency of 20 Hz. Level 10 of EMS training intensity was selected from twenty EMS in situ training levels. HPLT and LPHT were performed on bicycle condition and EMS-combined bicycle condition. Therefore, four types of conditions were conducted to each subject. Each condition was conducted at least 24 h apart to prevent residual muscle fatigue. Subjects are instructed to maintain a consistent power output by watching the monitor, which is synchronized with the sensor on the bicycle. The current pedal rate is displayed on the screen in real time, enabling subjects to match and maintain the same cycling rhythm throughout the exercise. EIT was measured before and after each power of bicycle training. Also, in order to verify the association between the spatial-mean conductivity and the blood lactate concentration, blood lactate concentration of the right earlobe was measured by lactate oxidase analyzer (Lactate Pro^TM^2, ARKRAY Inc., Japan) before and after each power of bicycle training. All experimental subjects gave written informed consent for the study according to the Committee for Human Experimentation of Chiba University (ethical code 29–13).

Fig. 2a shows the muscle compartments of the right thigh. The thigh muscles were divided into three muscle compartments, which are T1 compartment, T2 compartment, and T3 compartment. The T1 compartment consists of the rectus femoris, vastus medialis, vastus intermedius, and vastus lateralis. The T2 compartment consists of the biceps femoris, semimembranosus, and semitendinosus. The T3 compartment consists of the adductor magnus, gracilis, and sartorius.

### Analysis Method and Evaluation Method

In order to quantitatively evaluate the conductive response of thigh muscle compartments under four conditions, spatial-mean conductivity in each muscle compartment was measured before and after each power of bicycle training. The spatial-mean conductivity changes $\Delta \langle {\sigma }_{P}\rangle$ is defined as

4 $$\begin{array}{c}\Delta \langle {\sigma }_{P}\rangle =\frac{\langle {\sigma }_{P}\rangle -\langle {\sigma }_{0}\rangle }{\langle {\sigma }_{0}\rangle }\#\end{array}$$

where $P=103, 152,\text{ or }194 [\mathrm{W}]$ is the power during bicycle training. The $\langle {\sigma }_{0}\rangle$ is the spatial-mean conductivity before bicycle training and the $\langle {\sigma }_{P}\rangle$ is the spatial-mean conductivity after bicycle training with power $P$.

In order to evaluate the differences of spatial-mean conductivity changes $\Delta \langle {\sigma }_{P}\rangle$ between bicycle-operation conditions, paired *t*-test was conducted between $\Delta \langle {\sigma }_{P}\rangle$ under HPLT and the $\Delta \langle {\sigma }_{P}\rangle$ under LPHT. This comparison was conducted separately for each muscle-stimulation condition. The test statistic for the paired-samples $t$-test, the $t$-value is expressed as

5 $$\begin{array}{c}t=\frac{{\overline{x}}_{diff}\sqrt{n}}{{S}_{diff}}\#\end{array}$$

where ${\overline{x}}_{diff}$ denotes the mean of the paired differences across the $n$ experimental subjects, and ${S}_{diff}$ represents the standard deviation of these differences. The significance level was set at $\alpha =0.05$ (two tailed). The critical $t$-value ${t}_{0.025}$ was used as the reference threshold for statistical significance. Because the same number of subjects was used for all paired comparisons, ${t}_{0.025}$ was identical across all tests. Statistical significance was assessed by comparing the absolute value of the calculated $t$-statistic with ${t}_{0.025}$ or, equivalently, by evaluating the corresponding $p$-value.

In order to evaluate the difference of spatial-mean conductivity changes $\Delta \langle {\sigma }_{P}\rangle$ between muscle-stimulation conditions, paired *t*-test was conducted between $\Delta \langle {\sigma }_{P}\rangle$ under bicycle condition and the $\Delta \langle {\sigma }_{P}\rangle$ under EMS-combined bicycle condition. This comparison was conducted separately for each bicycle-operation condition. The significance level was set at $\alpha =0.05$ (two tailed).

Multiple linear regression analysis was conducted to investigate the factors contributing to the spatial-mean conductivity changes $\Delta \langle {\sigma }_{P}\rangle$. The dependent variable was $\Delta \langle {\sigma }_{P}\rangle$ and four predictors were included as independent variables: the power $P$ of the bicycle training (103, 152, and 194 $[\mathrm{W}]$), bicycle-operation conditions (HPLT, LPHT; reference: HPLT), muscle-stimulation conditions (bicycle condition, EMS-combined bicycle condition; reference: bicycle condition), and thigh muscle compartments (T1 compartment, T2 compartment, T3 compartment; reference: T1 compartment). Power $P$ was treated as a continuous variable, such that its regression coefficient represents the change in $\Delta \langle {\sigma }_{P}\rangle$ per 1 [W] increase in power. Categorical variables were dummy-coded, with reference levels selected based on physiological relevance and baseline condition. Regression coefficients $\beta$, standard errors, $t$-values, and $p$-values were reported for each predictor. Statistical significance was set at $p<0.05$. All analyses were performed using Python (statsmodels).

In order to verify the association between the spatial-mean conductivity and the blood lactate concentration, blood lactate concentration ${c}_{P}$ was measured before and after each power of bicycle training. Also, blood lactate concentration changes ${\Delta c}_{P}$ is defined as

6 $$\begin{array}{c}{\Delta c}_{P}=\frac{{c}_{P}-{c}_{0}}{{c}_{0}}\#\end{array}$$

where ${c}_{0}$ is the blood lactate concentration before bicycle training and ${c}_{P}$ is the blood lactate concentration after bicycle training with power $P$.

In order to compare the spatial-mean conductivity changes $\Delta \langle {\sigma }_{P}\rangle$ with the blood lactate concentration changes ${\Delta c}_{P}$, correlation coefficients $R$ between ${\Delta c}_{P}$ and $\Delta \langle {\sigma }_{P}\rangle$ was calculated. The $R$ is expressed as

7 $$\begin{array}{c}R=\frac{\sum \left(\Delta {c}_{P}-\overline{\Delta {c }_{P}}\right)\left(\Delta \langle {\sigma }_{P}\rangle -\overline{\Delta \langle {\sigma }_{P}\rangle }\right)}{\sqrt{\sum {\left(\Delta {c}_{P}-\overline{\Delta {c }_{P}}\right)}^{2}\sum {\left(\Delta \langle {\sigma }_{P}\rangle -\overline{\Delta \langle {\sigma }_{P}\rangle }\right)}^{2}}}\#\end{array}$$

where $\overline{\Delta {c }_{P}}$ and $\overline{\Delta \langle {\sigma }_{P}\rangle }$ are the averages of $\Delta {c}_{P}$ and $\Delta \langle {\sigma }_{P}\rangle$ under each condition. Also, in order to assess the statistical significance of each $R$, $t$-test was performed for each $R$. In order to evaluate the differences of the $R$ between HPLT and LPHT, Fisher's *z*-transformation was applied to each $R$, and paired *t*-tests were subsequently performed on the transformed values for both muscle-stimulation conditions. The significance level was set at $\alpha =0.05$ (two tailed).

## Results

### Conductivity Distribution Images of the Thigh Muscle Compartment

**Fig. 4:**
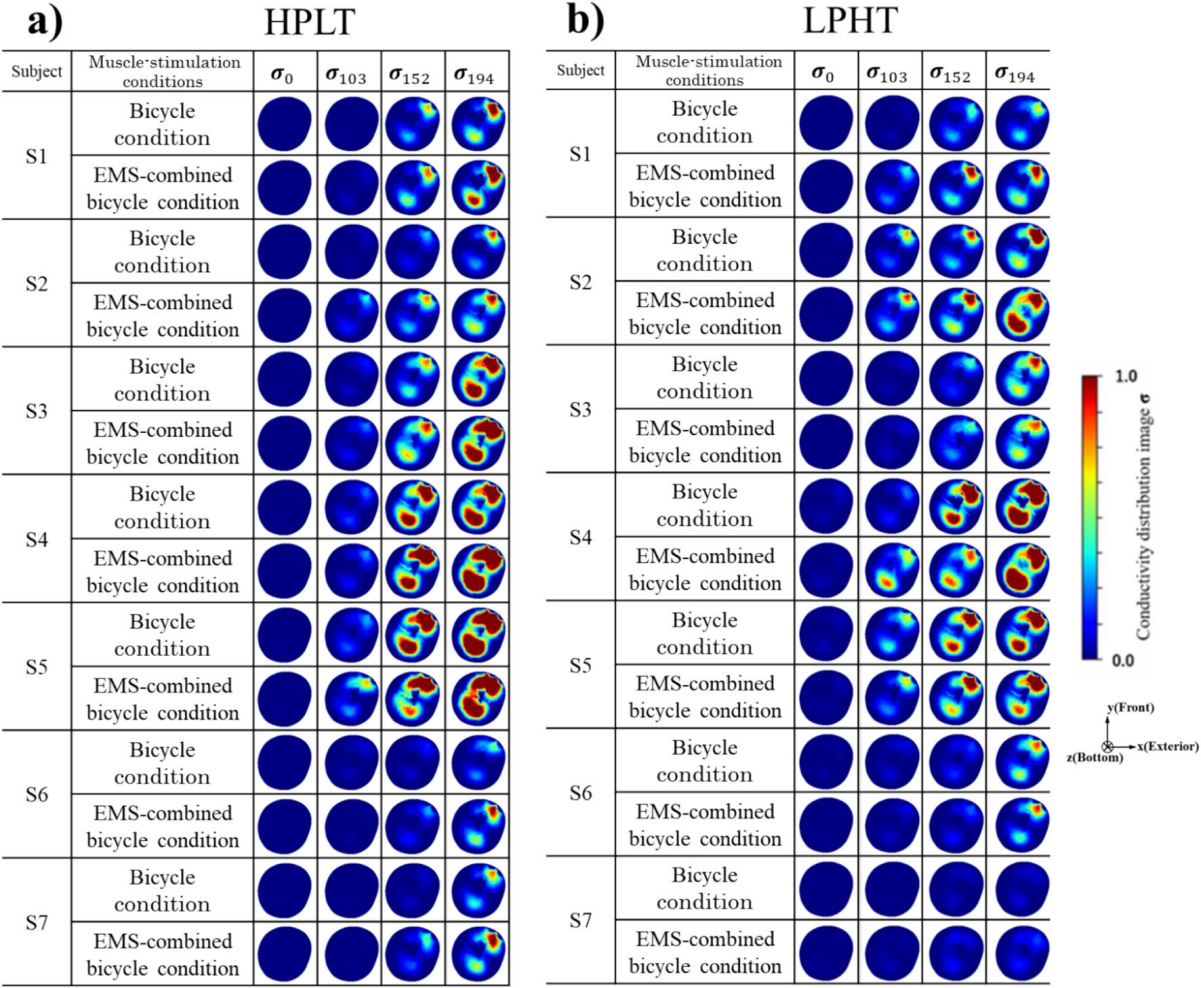
Conductivity distribution images ${{\boldsymbol{\sigma}}}_{P}$ under **a** HPLT. **b** LPHT

Fig. 4 shows the conductivity distribution images ${{\boldsymbol{\sigma}}}_{P}$ of the right thigh of seven subjects. The ${{\boldsymbol{\sigma}}}_{0}$ is the conductivity distribution images before the bicycle training. The ${{\boldsymbol{\sigma}}}_{P}$ is the conductivity distribution images after bicycle training with power $P[\mathrm{W}]$. The conductive responses are clearly imaged for all subjects in the T1, T2, and T3 compartments at ${{\boldsymbol{\sigma}}}_{152}$ and ${{\boldsymbol{\sigma}}}_{194}$ under HPLT, as shown in Fig. 4a. On the other hand, the conductive responses are clearly imaged for all subjects in the T1, T2, and T3 compartments at ${{\boldsymbol{\sigma}}}_{194}$ under LPHT, as shown in Fig. 4b. As power $P$ gradually increases, ${{\boldsymbol{\sigma}}}_{P}$ of the thigh muscle compartment becomes more visible. From the ${{\boldsymbol{\sigma}}}_{P}$ in general, the conductivity distribution between ${{\boldsymbol{\sigma}}}_{0}$ and ${{\boldsymbol{\sigma}}}_{194}$ under four conditions is clearly imaged, which shows that the tendency of conductive response is increased in all four conditions with increasing power $P$.

### Spatial-Mean Conductivity Changes of the Thigh Muscle Compartment

**Fig. 5:**
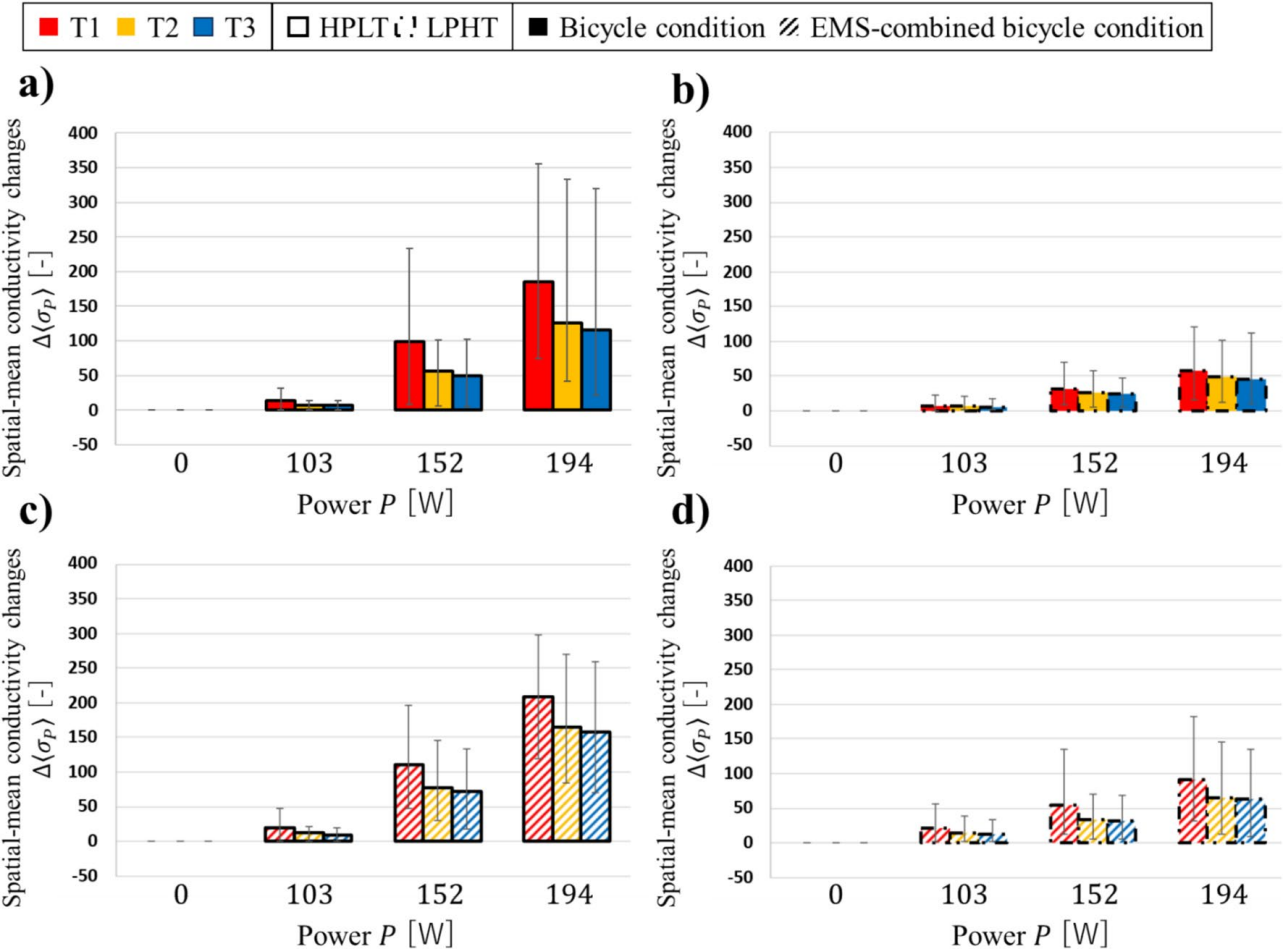
The spatial-mean conductivity changes $\Delta \langle {\sigma }_{P}\rangle$ of seven subjects **a** under HPLT bicycle condition. **b** under LPHT bicycle condition. **c** under HPLT EMS-combined bicycle condition. **d** under LPHT EMS-combined bicycle condition

Fig. 5 shows the spatial-mean conductivity changes $\Delta \langle {\sigma }_{P}\rangle$ of seven subjects under four conditions in three thigh muscle compartments. The error bars represent the maximum and minimum of the $\Delta \langle {\sigma }_{P}\rangle$ among seven subjects. The $\Delta \langle {\sigma }_{P}\rangle$ increased with increasing power $P$ of the bicycle training across all four conditions and in all muscle compartments ($n=7, p<0.05$).

Table 1 shows the results of the paired-samples $t$-test conducted between $\Delta \langle {\sigma }_{P}\rangle$ under HPLT and the $\Delta \langle {\sigma }_{P}\rangle$ under LPHT. This comparison was conducted separately for each muscle-stimulation condition. The ${t}_{0.025}(6)$ represents the critical value of the two tailed $t$ distribution at a significance level of 0.05 with 6 degrees of freedom. Because the same number of subjects and identical statistical criteria were applied in all analyses, this value is identical across all comparisons. Across all muscle compartments, the $\Delta \langle {\sigma }_{P}\rangle$ under HPLT tended to be higher than the $\Delta \langle {\sigma }_{P}\rangle$ under LPHT. In particular, $\Delta \langle {\sigma }_{194}\rangle$ under HPLT was significantly higher than $\Delta \langle {\sigma }_{194}\rangle$ under LPHT ($\left|t\right|>{t}_{0.025}\left(6\right), p<0.05$).

**Table 1:** Results of the paired-samples *t*-test conducted between $\Delta \langle {\sigma }_{P}\rangle$ under HPLT and the $\Delta \langle {\sigma }_{P}\rangle$ under LPHT for both muscle-stimulation condition

Power *P* [W]	Items	T1 compartment		T2 compartment		T3 compartment	
		Bicycle condition	EMS-combined bicycle condition	Bicycle condition	EMS-combined bicycle condition	Bicycle condition	EMS-combined bicycle condition
103	Mean difference	− 6.485	1.969	− 1.267	3.136	− 1.061	− 3.591
	Standard deviation	4.483	8.967	3.252	4.382	2.691	4.040
	${t}_{0.025}(6)$	2.447					
	$t$-value	− 1.447	0.2196	− 0.3896	− 0.7156	− 0.3945	− 0.8889
	$p$-value	0.1981	0.8335	0.7103	0.5011	0.7069	0.4083
152	Mean difference	**− 67.26**	− 57.25	− 31.07	**− 42.89**	− 25.58	**− 40.02**
	Standard deviation	**26.92**	23.49	14.23	**14.77**	13.56	**14.04**
	${t}_{0.025}(6)$	2.447					
	$t$-value	**− 2.499**	− 2.438	− 2.183	**− 2.904**	− 1.886	**− 2.850**
	$p$-value	** < 0.05**	0.05063	0.07176	** < 0.05**	0.1082	** < 0.05**
194	Mean difference	**− 127.9**	**− 117.8**	**− 77.87**	**− 99.49**	− 70.33	**− 94.93**
	Standard deviation	**34.64**	**19.52**	**30.19**	**25.36**	29.64	**26.15**
	${t}_{0.025}(6)$	2.447					
	$t$-value	**− 3.692**	**− 6.036**	**− 2.579**	**− 3.923**	− 2.373	**− 3.630**
	$p$-value	** < 0.05**	** < 0.01**	** < 0.05**	** < 0.01**	0.05529	** < 0.05**

Bold values indicate statistically significant results ($p<0.05$).

Table 2 shows the results of the paired-samples $t$-test conducted between $\Delta \langle {\sigma }_{P}\rangle$ under bicycle condition and the $\Delta \langle {\sigma }_{P}\rangle$ under EMS-combined bicycle condition. This comparison was conducted separately for each bicycle-operation condition. Although the difference was not statistically significant, the $\Delta \langle {\sigma }_{P}\rangle$ under EMS-combined bicycle condition tended to be higher than the $\Delta \langle {\sigma }_{P}\rangle$ under bicycle condition.

**Table 2:** Results of a paired-samples *t*-test conducted between $\Delta \langle {\sigma }_{P}\rangle$ under bicycle condition and the $\Delta \langle {\sigma }_{P}\rangle$ under EMS-combined bicycle condition for both muscle-stimulation conditions

Power *P* [W]	Items	T1 compartment		T2 compartment		T3 compartment	
		HPLT	LPHT	HPLT	LPHT	HPLT	LPHT
103	Mean difference	5.525	13.98	4.422	8.825	3.203	7.855
	Standard deviation	4.278	6.520	2.535	4.569	2.221	4.182
	${t}_{0.025}(6)$	2.447					
	$t$-value	1.291	2.144	1.745	1.931	1.442	1.879
	$p$-value	0.2441	0.0754	0.1317	0.1016	0.1994	0.1094
152	Mean difference	12.35	22.36	19.89	8.071	**22.24**	7.801
	Standard deviation	21.60	9.861	11.67	5.843	**7.755**	6.591
	${t}_{0.025}(6)$	2.447					
	$t$-value	0.5716	2.267	1.704	1.381	**2.868**	1.184
	$p$-value	0.5884	0.06393	0.1393	0.2164	** < 0.05**	0.2814
194	Mean difference	23.77	**33.83**	38.20	**16.59**	42.10	**17.50**
	Standard deviation	32.68	**8.008**	33.46	**6.210**	26.14	**7.084**
	${t}_{0.025}(6)$	2.447					
	$t$-value	0.7276	**4.225**	1.142	**2.671**	1.611	**2.471**
	$p$-value	0.4943	** < 0.01**	0.2971	** < 0.05**	0.1584	** < 0.05**

Bold values indicate statistically significant results ($p<0.05$)

## Discussion

### Multiple Linear Regression Analysis on each Independent Variable

**Fig. 6:**
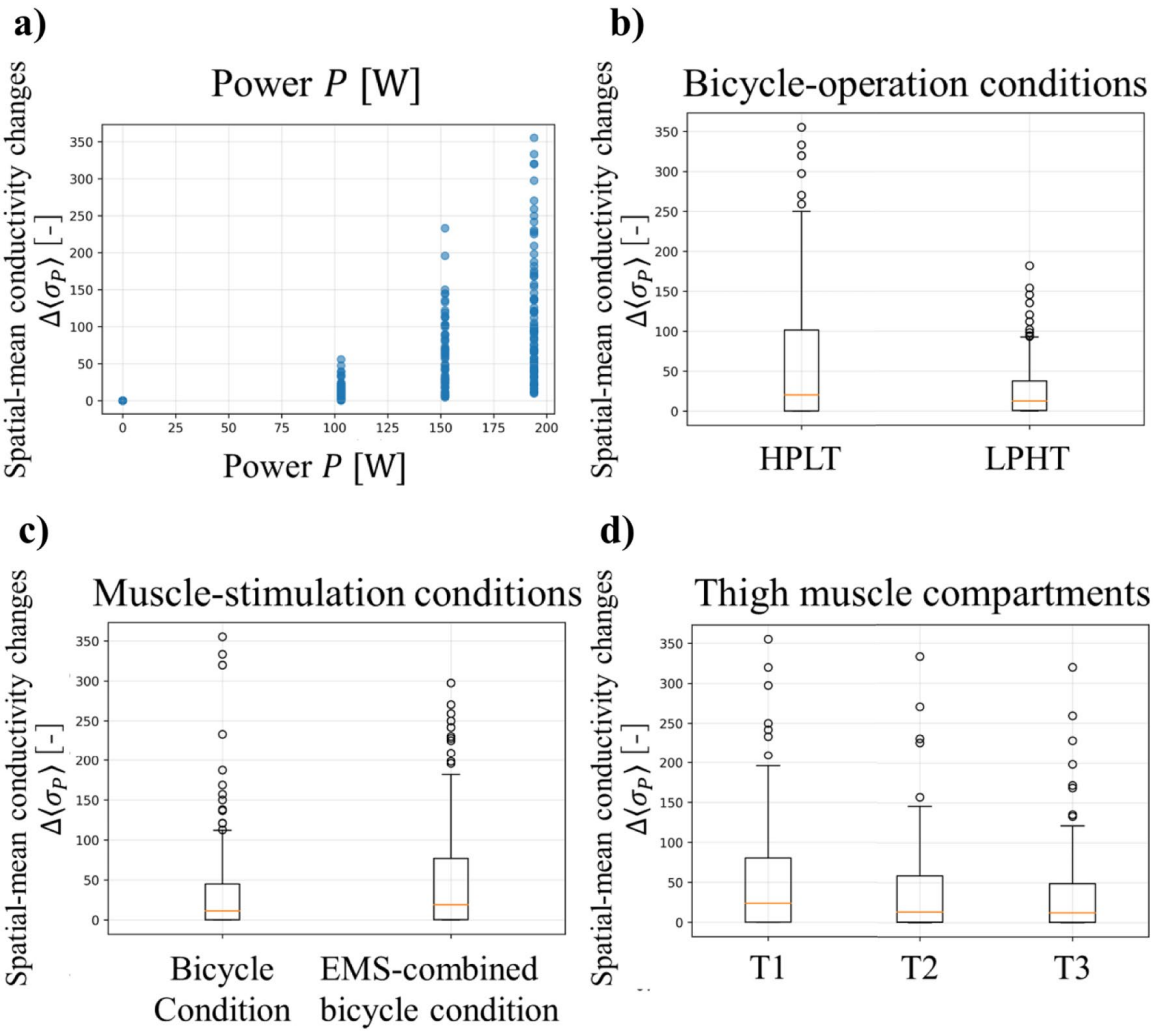
Associations between independent variables and spatial-mean conductivity changes $\Delta \langle {\sigma }_{P}\rangle$: **a** Power $P$, **b** bicycle-operation conditions, **c** muscle-stimulation conditions, and **d** thigh muscle compartments. Box plot show median, quartiles, and outliers for seven subjects

Fig. 6 shows the associations between the independent variables and the spatial-mean conductivity changes $\Delta \langle {\sigma }_{P}\rangle$. The independent variables include power $P$, bicycle-operation conditions, muscle-stimulation conditions, and thigh muscle compartments. Power $P$ demonstrates a strong positive linear relationship with $\Delta \langle {\sigma }_{P}\rangle$ as shown in Fig. 6a. It has been demonstrated that each watt increase contributes significantly to muscle activation which in turn drives $\Delta \langle {\sigma }_{P}\rangle$. Higher power intensities produce proportionally greater local metabolic demands and systemic physiological responses. Evidence for this relationship is demonstrated by increased lactate accumulation and muscle oxygen consumption. Furthermore, the power output directly determines metabolic stress and the requirements for muscle blood flow [40]. In bicycle-operation conditions, HPLT produce conductivity values that are twice as great as those produced by LPHT, as shown in Fig. 6b. It has been demonstrated that high pedaling rates in conjunction with low torque levels optimize the velocity of muscle fascicle shortening for the purpose of power production [41]. This is achieved by reducing intramuscular pressure, thereby facilitating enhanced blood perfusion and muscle pump action in comparison to low cadence/high torque conditions [42]. The EMS-combined bicycle condition has been shown to produce slightly higher median $\Delta \langle {\sigma }_{P}\rangle$ than bicycle condition alone, as shown in Fig. 6c. Thigh muscle compartment analysis demonstrated that the T1 compartment exhibits the highest $\Delta \langle {\sigma }_{P}\rangle$, followed by lower responses in T2 and T3 compartments, as shown in Fig. 6d. The quadriceps muscle serves as the primary force generator during the pedaling downstroke, making it the dominant muscle group for power production during cycling exercise [43].

Table 3 shows the result of multiple linear regression analysis that was performed on each independent variable that contributes to $\Delta \langle {\sigma }_{P}\rangle$. The multiple regression analysis quantified these relationships, confirming power as the strongest predictor ($\beta =0.531, p<0.001$), followed by the substantial negative effect of LPHT compared to HPLT conditions ($\beta =-35.52, p<0.001$), significant compartment differences with T2 and T3 showing reductions of 16.31 and 19.21 units respectively compared to T1 (both $p<0.05$), and a modest but significant EMS enhancement effect ($\beta =12.85, p<0.05$).

**Table 3:** The results of multiple linear regression analysis on each independent variable that contributes to $\Delta \langle {\sigma }_{P}\rangle$

Predictor	Coefficient $\beta$	Standard error	$t$-value	$p$-value
Intercept	7.896	7.320	1.079	0.281
Power *P* [W]	0.5310	0.037	14.29	< 0.001
Muscle-stimulation conditions (bicycle condition)†	12.85	5.379	2.390	< 0.05
Bicycle-operation conditions (HPLT)†	− 35.52	5.379	− 6.604	< 0.001
T2 compartment (T1 compartment)†	− 16.31	6.588	− 2.476	< 0.05
T3 compartment (T1 compartment)†	− 19.21	6.588	− 2.916	< 0.01

The symbol † indicates the reference

### Blood Lactate Concentration

**Fig. 7:**
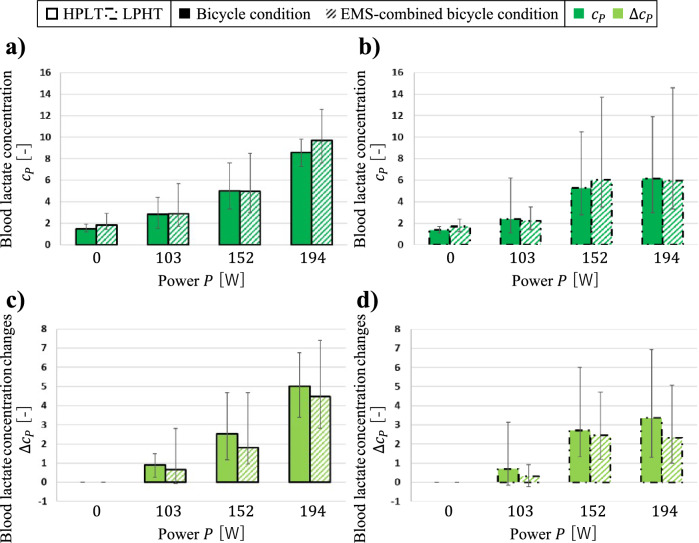
The blood lactate concentration ${c}_{P}$ of seven subjects **a** under HPLT. **b** under LPHT and the blood lactate concentration changes ${\Delta c}_{P}$ of seven subjects **c** under HPLT. **d** under LPHT

The spatial-mean conductivity changes $\Delta \langle {\sigma }_{P}\rangle$ may be associated with two main factors. The first factor is the accumulation of high concentrations of metabolites, such as lactate and H⁺ ions, within muscle cells [18]. Muscle contraction and relaxation require ATP. Under anaerobic conditions, glucose is primarily converted into lactate via the glycolytic pathway to produce ATP [44]. As the intensity of muscle contraction increases, the demand for ATP also rises, resulting in increased lactate production through glycolysis [18]. A previous study using electrical impedance myography (EIM) indicated that resistance decreased post-fatigue compared to the beginning of contraction, which may reflect metabolite accumulation in fatigued muscle tissue [45]. The second factor is the increase in blood plasma volume in the interstitial spaces surrounding muscle cells [18]. Muscle contraction occludes the veins of the contracted muscle, meaning that a large amount of blood enters the muscle through the arteries while only a small amount exits through the veins. This leads to an accumulation of blood plasma in the interstitial spaces around the muscle cells [18]. This finding is consistent with a study using bioimpedance spectroscopy (BIS), which reported a region-specific reduction in extracellular resistance following eccentric exercise of the elbow flexors, likely attributed to swelling [46]. In order to verify the potential association between $\Delta \langle {\sigma }_{P}\rangle$ and the metabolic factors, blood lactate concentration was measured before and after bicycle training with power $P [\mathrm{W}]$. Fig. 7 shows the blood lactate concentration ${c}_{P}$ of seven subjects and the blood lactate concentration changes ${\Delta c}_{P}$ of seven subjects under four conditions. The error bars represent the maximum and minimum of the ${c}_{P}$ and ${\Delta c}_{P}$ among seven subjects. Although the difference was not statistically significant, the ${c}_{P}$ and ${\Delta c}_{P}$ under HPLT tended to be larger than the ${c}_{P}$ and ${\Delta c}_{P}$ under LPHT. Additionally, there was no significant difference between ${c}_{P}$ and ${\Delta c}_{P}$ under bicycle condition and the ${c}_{P}$ and ${\Delta c}_{P}$ under EMS-combined bicycle condition. These findings suggest potential associations between ${\Delta c}_{P}$ and $\Delta \langle {\sigma }_{P}\rangle$, though the specific physiological mechanisms underlying the $\Delta \langle {\sigma }_{P}\rangle$ remain to be determined.

### Comparison Between Bicycle-Operation Conditions

The blood lactate concentration changes ${\Delta c}_{P}$ under HPLT tended to be larger than the ${\Delta c}_{P}$ under LPHT. Additionally, previous research has shown that the number of muscle contractions under HPLT is greater than the number of muscle contractions under LPHT [5]. These factors may contribute to differences in blood plasma volume in the interstitial spaces surrounding muscle cells between HPLT and LPHT conditions [5], though the present study did not directly measure these physiological parameters. Thus, HPLT is a more efficient bicycle training than LPHT for increasing $\Delta \langle {\sigma }_{P}\rangle$**.**

### Comparison Between Muscle-Stimulation Conditions

There was no significant difference between the blood lactate concentration changes ${\Delta c}_{P}$ under bicycle condition and the ${\Delta c}_{P}$ under EMS-combined bicycle condition. However, the $\Delta \langle {\sigma }_{P}\rangle$ under EMS-combined bicycle condition tended to be larger than the $\Delta \langle {\sigma }_{P}\rangle$ under bicycle condition in our measurements. Previous studies have shown that EMS rapidly activates muscle fibers, even at low force levels [47], triggering an immediate physiological response that leads to changes in the muscle’s extracellular water content [9]. These findings suggest that the larger $\Delta \langle {\sigma }_{P}\rangle$ observed might be associated with the increase in blood plasma volume in the interstitial spaces surrounding muscle cells. Therefore, EMS-combined bicycle condition is a more efficient bicycle training than bicycle condition for increasing $\Delta \langle {\sigma }_{P}\rangle$.

### Correlation Between Blood Lactate Concentration Changes and Spatial-Mean Conductivity Changes

**Fig. 8:**
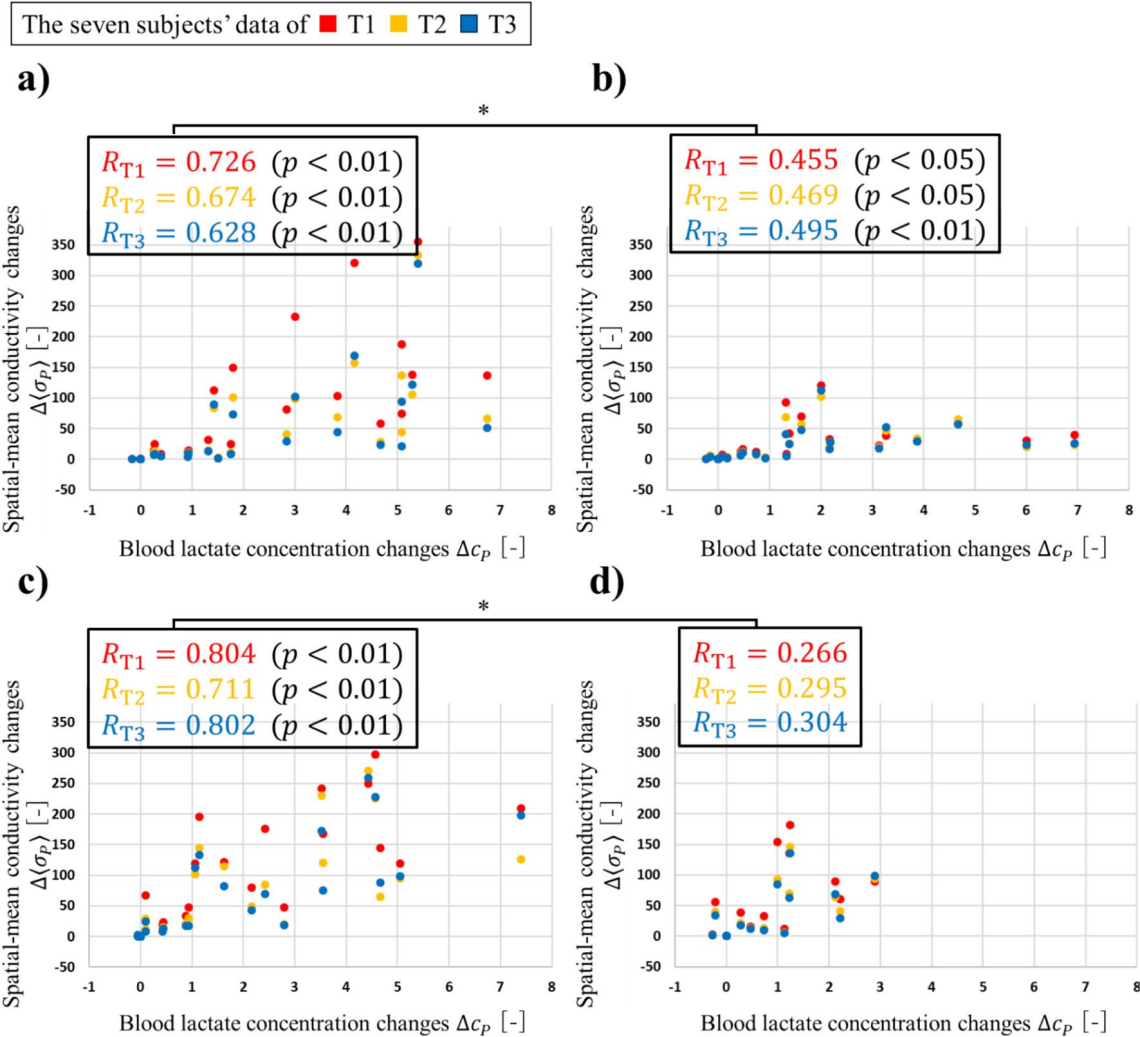
Correlation between the blood lactate concentration changes $${\Delta c}_{P}$$ and the spatial-mean conductivity changes $$\Delta \langle {\sigma }_{P}\rangle$$ under **a** HPLT bicycle condition. **b** LPHT bicycle condition. **c** HPLT EMS-combined bicycle condition. **d** LPHT EMS-combined bicycle condition. $${}^{*}p<0.05, {}^{**}p<0.01$$

Fig. 8 shows the correlation between the blood lactate concentration changes ${\Delta c}_{P}$ and the spatial-mean conductivity changes $\Delta \langle {\sigma }_{P}\rangle$ under four conditions. Also, Fig. 8 shows the correlation coefficient $R$ and presents the results of a *t*-test assessing the statistical significance of each $R$. Additionally, Fig. 8 shows the results of a paired-samples *t*-test conducted between $R$ under HPLT and the $R$ under LPHT for both muscle-stimulation conditions after Fisher's *z*-transformation was applied to each $R$. The correlation under HPLT was strongly positive, and the correlation under LPHT was slightly to moderately positive for both muscle-stimulation conditions. Since the correlation was positive under all four conditions, it could be suggested that EIT imaged the conductive response associated with blood lactate concentration under bicycle-operation conditions and muscle-stimulation conditions. The $R$ under HPLT was significantly higher than the $R$ under LPHT for both muscle-stimulation conditions. One possible reason for this is that, although the difference is not statistically significant, ${\Delta c}_{P}$ tends to increase gradually under HPLT between ${\Delta c}_{152}$ and ${\Delta c}_{194}$, whereas under LPHT, ${\Delta c}_{P}$ shows only a slight increase or decrease over the same range. However, the underlying mechanism behind this observation remains unclear and warrants further investigation in future studies.

One limitation of this study is the small and homogeneous sample, consisting of only seven healthy men (age: 27.5 ± 6.5 years, height: 175.0 ± 8.0 cm, skeletal muscle mass: 28.6 ± 6.5 kg). The restricted sample size, narrow age range, exclusion of women, and focus on a single ethnic group limit the generalizability of the findings. Another limitation is the use of a single thigh model that did not account for individual thigh size differences or changes during exercise, which may have affected the accuracy of the impedance measurements. Temperature and sweat effects also could not be entirely excluded, although sweat was wiped off after each bicycle training with power $P [\mathrm{W}]$.

The Gaussian-Newton method employed in this study is well-established; however, recent developments in EIT, including sparsity-based regularization, deep neural networks, and hybrids, which may provide improved spatial resolution and could potentially reduce artefacts [48]. These advanced solvers might be valuable for capturing subtle, exercise-induced conductivity changes and for minimizing image artefacts during intensive exercise protocols.

Future studies should include larger, more diverse cohorts including women, wider age ranges, different ethnic groups, and potentially patients with muscle disorder. The robustness of EIT for muscle conductivity measurement will be evaluated in a range of physiological and pathological conditions.

The present study reveals that the measured impedance changes in muscle areas of human thigh under bicycle-operation conditions and muscle-stimulation conditions have been detected by EIT. The key findings of this study are as follows. EIT imaged the conductive response that were associated with blood lactate concentration under bicycle-operation conditions and muscle-stimulation conditions. The spatial-mean conductivity changes $\Delta \langle {\sigma }_{P}\rangle$ increased with increasing power $P$ of the bicycle training across all four conditions and in all muscle compartments. HPLT is a more efficient bicycle training than LPHT for increasing $\Delta \langle {\sigma }_{P}\rangle$**.** EMS-combined bicycle condition is a more efficient bicycle training than bicycle condition for increasing $\Delta \langle {\sigma }_{P}\rangle$**.**

## Data Availability

The data that supports the findings of this study are not openly available for study participant privacy reasons. They are available from the corresponding author upon reasonable request.
